# A Treatise on the Diseases of the Breast and Mammary Region

**Published:** 1857-03

**Authors:** 


ART. II.—*A Treatise on the Diseases of the Breast and Mammary Region.* By A. VELPEAU, Membre de l'Institut, Professeur a la Faculté de Médecine de Paris, Chirurgien de l'Hôpital de la Charité, &c. Translated by MITCHELL HENRY, Fellow by examination of the Royal College of Surgeons, &c. &c. London, 1856. Printed for the Sydenham Society.

*A Treatise on Cancer of the Breast and of the Mammary Region.* By A. VELPEAU, Member, &c. Translated from the French by W. MARSDEN, M.D., Surgeon to the Royal Free and Cancer Hospital. London. Renshaw, 1856.

THE treatise before us, from the pen of the great French clinical professor, is devoted to the consideration of the diseases of the breast and of the mammary region. The original of this work appeared in Paris in the early part of the year 1854, and attracted, at that time, to a very considerable degree, the attention of the surgical public. It embodies the vast experience of one who, for more than thirty years, has occupied a position deservedly high in his profession, and whose opportunities for observation have been rarely, if ever, equalled. M. Velpeau combines the characteristics of a scientific investigator with those of a great practical surgeon; and we would therefore naturally suppose that any treatise emanating from such a source could not but be destined to occupy a lasting place in the literature of the profession. The very fact of the translation of this work into English having been undertaken by two writers, one of them under the auspices of the Council of the Sydenham Society, seems in itself sufficient testimonial to its excellence.

In the Author's Preface, we are informed that the treatise under consideration is designed to partially fill a vacuum which now exists in French practice concerning diseases of the mamma. The work of Sir Astley Cooper, and the articles of Boyer and others, are evidently looked upon as obsolete; and the present monograph, based upon a study of more than two thousand original cases, is intended to supersede them. Whether or not such will be the case we leave it to our readers to decide.

At the very commencement of the work, the author draws the distinction between innocent or non-malignant, and malignant diseases; and the first section, constituting more than one half of the volume, is occupied with the consideration of the former.

Innocent or benign diseases are defined to be "those which, left to themselves, do not threaten the patient's life, and of which recovery is the natural termination." These are then divided into two groups: 1st, those in which the morbid process is due to the existence of the different kinds of inflammation; and 2d, those which in their access or duration, are strangers to the inflammatory process.

Under the head of inflammatory affections are included excoriations, chaps, fissures, eczema, erysipelas, milk knots, and all the varieties of phlegmon; and it is stated that many of these affections seem to exert a kind of preference in the selection of their site. Some attack the nipple; some the areola, or lactiferous tubes; whilst others affect almost exclusively the gland alone, or its investment. These diseases may, however, occur simultaneously, or spread by contiguity from one tissue to others adjoining.

*Mammitis, mastitis*, or inflammation of the breast, properly so called, M. Velpeau next proceeds to discuss. His arrangement of this disease is the one which he first proposed in 1825, and which has since been adopted by MM. Nelaton, Berard, and Vidal. This division is founded upon the exact anatomical seat of the inflammatory process, and is insisted upon somewhat decidedly by the author, in consequence of the different treatment which he considers proper under each circumstance. The first form of inflammation is that which occurs in the subcutaneous cellulo-fatty substance which is situated between the gland and the skin. The second variety is seated either primarily or secondarily beneath the breast, between the gland and the walls of the chest; whilst the third and last variety occupies the substance of the gland itself. In tabular form they may be thus expressed:

| | | |
|---|---|---|
| Phlegmon. | 1. Inflammation, superficial or *subcutaneous*, | Circumscribed.<br>Diffuse.<br>Primary.<br>Secondary.<br>Complex. |
| | 2. Inflammation, deep-seated or *submammary*, | Idiopathic.<br>Symptomatic.<br>General or diffuse.<br>Partial or circumscribed.<br>Primary or secondary. |
| | Lactiferous Engorgement. | |
| | 3. Inflammation, glandular or *parenchymatous*, | Of the milk tubes.<br>Of the glandular tissue.<br>Complex. |

We will not weary our readers by entering into a minute analysis of the above subvarieties of inflammation, but content ourselves by merely pointing out the general differential diagnosis between the three main forms of the affection.

The peculiar character of subcutaneous inflammation of the breast seems to be dependent upon the anatomical arrangement of the tissues. M. Velpeau states that the skin of the breast is separated from the deeper structures by a layer of cellulo-fatty substance, which forms a portion of the general superficial fascia. This layer passes outwards, and becomes continuous with the subcutaneous fascia of the chest and axilla, but is nowhere reflected between the breast and the walls of the chest. It is thinnest in the neighborhood of the areola, at which point it blends with the skin, and with the capsule of the gland.

It follows from this arrangement, that inflammation of this tissue is lighter in its character and of less consequence as it approaches the nipple; and, moreover, that there exists but little tendency in abscesses forming in this region to burrow in the neighborhood of the deeper-seated parts.

The whole subject of mammitis is thus summed up:

"Idiopathic subcutaneous inflammation arises in the breast under the influence of the same causes as in all other parts of the body. Deep-seated inflammation may proceed from external violence, or from affections of the chest or axilla, but usually originates in some affection of the breast itself.

As for inflammation of the gland-structure, it is obvious that lactation, confinement, or pregnancy, constitute almost its exclusive sources."

The symptoms of the subcutaneous variety are: "Those of inflammation, being characterized by redness, circumscribed or diffuse tumefaction, which is elevated above the surface of the integuments, and accompanied by a sort of œdema or puffiness, the whole being essentially different from that form of inflammation beneath the breast, which manifests itself from the commencement by a raising up of the whole mamma, and often remains till the end without giving rise to well-marked redness, or to manifest irregularities on the surface of the part. Neither can we confound this inflammation with that in the glandular structure, which shows itself primarily in the form of bosses more or less deep and numerous, and is preceded or complicated with suppression or retention of the milk, and often involves several parts of the breast at the same time.

Subcutaneous inflammation, like ordinary phlegmon, lasts little more than a week before it terminates in abscess, in abscess which usually remains single, and seldom, when formed, escapes the attention of the practitioner. Deep-seated inflammation, on the contrary, although perhaps developing itself more rapidly, presents this remarkable feature,—that suppuration, if it take place, cannot be recognized until considerably later.

Parenchymatous inflammation is almost always constituted of several successive inflammations, so that in some instances it may last for one, two, or three months.

So marked a difference in the symptoms and progress of the cases naturally entails noticeable differences in their termination and prognosis. When energetically attacked from the commencement, both superficial and deep-seated inflammation may sometimes be put an end to, and terminate in resolution. In the gland itself, inflammation, if it be at all acute, almost inevitably leads to the formation of one or more abscesses.

As for the treatment, it has every chance of succeeding, if it be appropriately selected for each kind of inflammation,—leeches in large number to the affected part, mercurial friction, compression. Topical remedies, generally useful, succeed well in subcutaneous phlegmon. They are, on the contrary, inadequate in deep-seated and also in parenchymatous inflammation. Deep-seated inflammation which is not benefited by them, and is aggravated by compression, requires general bleeding, with leeches around the breast, and large poultices with mercurial ointment. It is in glandular inflammation that purgatives, alterative drinks, and topical applications, purely emollient in character, are useful. Ammoniacal, camphorated, and opiate liniments, are equally applicable to lactiferous engorgements.

When, in short, we find that, in spite of all our treatment, mammary adenitis often lasts, by becoming extended, a considerable number of weeks, whilst under appropriate treatment subcutaneous and submammary inflammation scarcely continue more than from a week to a fortnight, the importance of the distinctions laid down above ceases to be questionable."

*Painful enlargement* of the breast, the result of *lactiferous engorgement* of the milk-tubes, is to be carefully distinguished from inflammation, in the following manner: In the first place, there is no inflammation in the surrounding tissues. 2d. If the normal state of fluidity of the milk be restored, the symptoms cease. 3d. It disappears from day to day under the influence of heat or stimulant applications. The treatment consists in emptying the engorged ducts, either by the mouth of the child, or else by artificial suction.

From what has been said relative to the seats of inflammation it follows, necessarily, that the resulting abscesses will occupy the same location. M. Velpeau, consequently, describes abscess of the breast under three forms: subcutaneous, submammary, and glandular.

The position of the first variety is most frequent, according to the experience of M. Velpeau, upon the external and inferior portion of the breast,—a fact which he attributes to the dependent position of the organ.

The *second* or submammary form is generally ushered in by considerable constitutional disturbance, shivering, and lancinating pains. The tendency of these abscesses is generally to open at some point in the circumference of the breast; hence the incision for the evacuation of its contents should generally be made in this region, and at the most dependent point.

A peculiar form of abscess described by the author is the *shirt-stud* abscess (*abcès en bouton de chemise*). In this variety the purulent matter extends from behind the gland forwards, following the septa of the breast, and eventually gives rise to the formation of one or more subcutaneous depots, which communicate with the original submammary abscess by means of the intervening sinus. The cure under these circumstances is best obtained, in the judgment of M. Velpeau, by following the practice of Mr. Hey, of Leeds, which consists in laying open the sinus throughout its entire extent by a free incision, and in the subsequent introduction of dossils of lint, so that the wound may granulate from the bottom.

The parenchymatous or true glandular abscess is, however, by far the most common variety, and is met with most frequently after confinement, and much more frequently in women who suckle than in those who do not. Thus, in the appended statistical résumé of more than 200 cases, we find that "the varieties of abscess were: subcutaneous, 37; submammary, 38; glandular, 95; not specified, 6. The causes were: contusions, 20 times; eczema, 3 times; pregnancy, 7 times; confinement, 110 times; cracks in nipple solely, 2 times; a needle impacted in breast, 1 time; chills, 6 times. As regards suckling: had nursed, 75; had not nursed, 4."

The chapter is terminated by the advocation of compression as a curative agent of acute inflammation of the breast. This treatment was openly advanced by the author in 1826, since which period he has frequently employed it. He now claims that "it is one of the best resources that we possess, in the case of simple, or as in the preceding cases of complicated subacute sucklings, accompanying or following abscess of the breast." At the same time, M. Velpeau admits that there are very many cases in which it can scarcely be employed at all, and others in which its application is too troublesome and difficult. When desirable, pressure can be best exerted by means of strips of adhesive plaster. To be effectual, it must be well done; badly applied, the condition of the patient will only be aggravated.

In Chapter II, the diseases of an innocent nature, not inflammatory, are considered.

*Ecchymosis.*—M. Velpeau recognizes with Sir Astley Cooper two varieties of this affection: 1. Spontaneous ecchymosis, occurring chiefly in unmarried girls, at or immediately preceding the catamenial period. This variety is referable chiefly to defective menstruation, and to the sympathetic relation subsisting between the uterus and mammary gland. 2. *Contusion*, properly so called, produced by external violence, causing vascular rupture. Injuries of this character not unfrequently prove the exciting causes of tumors, malignant or otherwise.

The earlier pages of the second section treat of the manifold *engorgements* of the breast. M. Velpeau, although objecting to this term, still thinks proper to retain it.

*Physiological* engorgement, he defines to be that change in the shape and size of the mamma occurring chiefly in young unmarried women at the approach of the menstrual period. It is not a disease, but disappears rapidly, and is dependent upon excitement of the genital and sexual functions.

*Simple engorgement* may result from blows, falls, and various irritations; or they may occur during the progress of pregnancy. Frequently, these engorgements depend upon the existence of subacute inflammation, and the treatment will be sufficiently indicated.

*Hypostatic engorgements*, dependent upon the weight and pendulous condition of the breast, are often met with, especially in women who have borne children, and whose breasts are soft, heavy, and pendulous; and also in those whose breasts are simply voluminous. The treatment consists in the application of a suspensory bandage, handkerchief, or adhesive strip.

The consideration of the different varieties of innocent tumors occupies the remaining pages of this section. Innocent or homologous tumors may be divided, according to M. Velpeau, into two classes: 1. Those which consist of solid materials: 2. Those containing liquid or pultaceous matter —in other words, concrete tumors or cysts. At the same time, the word cyst is not to be too strictly interpreted, for it must be borne in mind, that solid tumors are often surrounded by a perfect sac.

Concrete tumors also admit of further subdivision, according as they consist either of exaggerated developments of normal tissues, or of new products. *Hypertrophies*, the first of these divisions, are then minutely considered; after which the author passes to the examination of lipomatæ, which may be mistaken for cystic tumors, as happened, we are informed, to himself.

*Induration of the mamma*, we are told, is of two kinds, subacute and chronic, with and without tumefaction or swelling. This section contains little that is new. We would, however, remind our readers that this induration has been made the subject of special investigation by Mr. Birkett, who tells us that the essential element of the disease consists in an excessive packing of epithelial cells in the cæcal terminations of the ducts, dependent doubtless upon some exalted action in the terminal cæci.

The pages devoted to neuromata or nodosities present us little that is interesting; the cases are unsatisfactory, and in no instance have the tumors been subjected to microscopical examination. Indeed, this fact strikes us most forcibly throughout the entire work, and we are often led to doubt the results placed before us, despite the self-vaunted skill of the author as a diagnostician.

The chapter on neuralgia of the breast appears to us as especially unfair. M. Velpeau claims to be the first to draw the attention of the profession to the existence of neuralgia of the breast, unaccompanied by the formation of tumors. Sir Astley Cooper, however, as early as 1829, distinctly described the characteristics of the disease.*

Under the head of *imaginary tumors*, M. Velpeau alludes to those frequent instances in which a nervous female, after the reception of some real or fancied injury, is led to believe in the growing development of a tumor. This belief is sometimes fostered by an injudicious examination on the part of the surgeon, or by the existence of abnormal peculiarities in the region hitherto unobserved. Thus, irregularity in the size and forms of the lobules of the gland, undue form and irregular projection of the ribs, may, if they be not properly interpreted, contribute to mislead the patient, and perhaps the surgeon. M. Velpeau quotes instances of this kind.

*Galactoceles*, lacteal swellings, or tumors, formed of milk, were originally described by Sir A. Cooper as arising from chronic inflammation and obliteration of one or more of the lactiferous tubes near the nipple. These same tumors are arranged, by our author, somewhat differently; thus: 1. Galactocele, from infiltration; 2. Liquid galactoceles, or lactiferous cysts; and,

* Diseases of the Breast, Am. Ed. p. 55.

3. Solid or concrete galactoceles, or butyrous tumors. In this latter form, M. Velpeau thinks that he has observed a tendency to return, and he asks himself the question, whether, in the primary operation, it would not be better to perform the ablation of the entire gland, rather than the mere removal of the tumor.

The consideration of *mammary cysts* and *hydatids* occupies the remaining thirty pages of this section. M. Velpeau is evidently disposed to question the observation of Sir Astley Cooper and Dr. Warren relative to the frequency of true hydatid cysts, dependent upon the presence of the parasitic echinococcus hominis. He avers, that he himself has never met with any such case; and he believes, that very frequently ordinary serous, or sero-sanguinolent cysts, have been mistaken for the variety in question. From the perusal of these pages, it seems to us, that the author is not sufficiently conversant with the views of the more recent English pathologists relative to mammary affections; or, even if he be, that he is inclined to lend them but little faith.

The terminal fifty pages of this section are devoted to the description of the pathology and treatment of a class of tumors, to which M. Velpeau applies the term "adenoid."

These growths were originally described by Sir Astley Cooper, under the name of "chronic mammary tumors"—an appellation, which, in the opinion of the author, is objectionable, since it may perhaps be suggestive of mammary inflammation. The term "adenoid" (glandlike), he considers to be preferable; being sufficiently descriptive of the external or naked-eye appearance of the tumor, and, at the same time, non-committal as to their histological character.

These tumors occur chiefly in unmarried females, or in women who have never borne children, and more especially in those under the age of thirty years. Their development is usually slow, and unattended by pain; but their chief characteristic is *mobility*. Pressure causes them to slide upon the surface of the gland; and, in so displacing them, we feel, at the same time, that no portion of the gland follows them. Our author informs us, that Sir Astley Cooper and Berard have erroneously supposed that these tumors were always, or nearly always, "subcutaneous," and superficial to the gland; and, he adds, that such is not the case, as he himself has found, on many occasions, that they occupy every portion of the depth and thickness of the breast. Upon this point, however, M. Velpeau has evidently misunderstood Sir Astley, who expressly states, that such a tumor generally appears to be superficial, "excepting if it spring from the posterior surface of the breast, when it is deep-seated, and its peculiar features are less easily discriminated."*

* Diseases of the Breast, Am. Ed. p. 39.

The pages devoted to the "Pathological anatomy of adenoid tumors," offer us but little that is new; indeed, we do not even find a recapitulation of what is already known upon the subject. A good deal is said concerning "granular," "cauliflower," or "pomegranate," appearances; but we find hardly any observations, or facts, upon which we can really rely. The labors of Lawrence, Birkett, Paget, Lebert, and others, are slurred over, if not entirely ignored, and the results furnished by the microscope are dismissed with a covert sneer.

The real structure of these tumors has already been almost entirely elucidated. Cooper describes them, "As if nature had formed an additional portion of the breast, composed of similar lobes;" and Mr. Birkett characterizes their microscopical structure as strikingly similar to that of an inactive mammary gland, as, for example, that of the male; and he is corroborated in his assertions by the studies of Paget and Lebert.

M. Velpeau looks upon these tumors as those of an essentially innocent nature; although, at the same time, he throws out the idea of a possible subsequent cancerous degeneration.

The treatment to be pursued is summed up thus: If the tumor is stationary, and the patient comfortable, let it alone; if the woman be of an irritable and anxious disposition, make use of resolvent applications and placebos. When, however, the anxiety of the patient is such as to prey upon her mind, advise surgical interference, telling her, at the same time, that there is no danger in the retention of the tumor, although there is small chance of a cure following the use of medical applications; and, moreover, that the tumor, once extirpated, cannot return.

We would remark, however, before closing our analysis of these tumors, that most often their early development is due to some sympathetic uterine or sexual influence; hence, it has not unfrequently happened, as in the instances recorded by Sir A. Cooper and our author, that marriage, pregnancy and, suckling had proved the means of effecting radical cures, —tumors, existing in one or both breasts, gradually subsiding, and never afterwards making their appearance.

The section on innocent tumors, 334 pages, closes with a table of "Adenoides, operated upon successfully by the author," from the year 1836 to 1853, inclusive. Total, 60 operations; not one of the patients died; all eventually recovered.

Diseases of a malignant nature, or cancers of the mammary region, are considered in the second section of the work.

Premising that "cancer of the mamma" differs neither in its nature, nor in its form, from cancer of other parts of the body, M. Velpeau offers us the following tabular division of the disease:

SCIRRHUS—

I. § 1. Ligneous scirrhus.

(*a*) Scirrhus, properly so called, or globular.

(*b*) Radiated or branched scirrhus.

(*c*) Cuirass-formed, or tegumentary scirrhus.

(*d*) Hard or ligneous scirrhus, "en masse."

(*e*) Atrophic scirrhus.

(*f*) Pustular, or disseminated scirrhus.

(*g*) Scirrhus of the lactiferous ducts.

§ 2. Lardaceous scirrhus.

II. Encephaloid.

III. Melanosis.

IV. Chondroid, colloid, fibro-plastic cancer.

1. Napiform tumors, or fibro-plastic, properly so called.
2. Colloid cancer.

V. Epithelial cancer, or epitheliome.

VI. Keloids.

VII. Anomalous cancer.

Under the denomination of "ligneous" or hard scirrhus, M. Velpeau includes a kind of tumor possessing the density and inextensibility of wood. These masses are without fixed limits, and are continuous, without appreciable lines of separation, into the neighboring tissues. It is during the course of this form of the disease, that the integuments covering the tumor are especially apt to become involved, acquiring a grayish tint, and that wrinkled, honeycombed appearance, which the writer conceives to be pathognomonic of hard cancer.

The *cuirass-formed* or tegumentary scirrhus, "scirrhus en cuirasse," is a variety of this affection, which is minutely described—commencing either in a single disc or at several isolated points in the integument of the breast. This morbid process may extend itself by gradually-increasing plates, until finally the whole anterior portion of the thorax may be plated or enveloped. Dissection reveals the seat of the affection to be exclusively the skin, which, sometimes greatly increased in thickness, may eventually acquire a density which is comparable only to the tanned hide of an animal. The early development of this form of scirrhus is insidious, so much so as to escape the attention of the patient, and even of the surgeon, unless he be on his guard. Two cases of this variety, the prognosis of which M. Velpeau considers of the most unfavorable kind, are reported in detail.

Under the term "scirrhus en masse," the author describes that form of cancer, in which the whole tissue of the mammary gland is involved almost simultaneously. This may occur, however, sometimes as a secondary form, following, for example, the cuirass-formed cancer before mentioned.

It is to be carefully distinguished from the second great group of carcinomatous disease, viz., the lardaceous cancer. In this latter form, the disease, although involving finally the greater portion of the gland, seems to commence by invading at first simply some of its more deeply-seated lobules.

Its density is sensibly less than that of the true ligneous scirrhus, and the integuments are, of all the tissues, the last involved.

Although M. Velpeau has drawn the lines of demarcation between the various forms of hard cancer, with great nicety, he, at the same time, informs us that he does not wish to be understood as assuming these varieties to be essentially distinct. He tells us that they all possess the same base, and are of the same nature. The general grounds of distinction upon which he most relies, are the hardness and density of the masses, and, the fact already noticed, that all of these tumors are, as it were, confounded with the surrounding natural tissues. He states, also, that he considers that there is not one of all these heterologous products, which is not formed by a degeneration, or transformation of the primitive anatomical element, rather than by a deposit of foreign matter. In this view, M. Velpeau stands at variance with many, indeed, most of the best English and German pathologists, who regard the cancerous matter as a new formation, deposited from the blood in infiltrating masses in the substance of tissues previously sound.

The *encephaloid* variety of cancer is treated of in the second article of this chapter. For the purposes of clinical study, M. Velpeau divides tumors of this nature into two classes, one of which he calls *fungous*, and the other, of a tolerably firm consistency, *lardaceous.*

These tumors commence by the formation of diseased masses at some distance beneath the integuments, and in their early stages present to the touch the feeling of knotted swellings. These swellings, with their subjacent indurated bases, are likened by the author to "the head of a cauliflower," buried deep in the principal masses.

The development of these tumors takes place with considerable rapidity, and the skin, instead of being thickened, indurated, wrinkled, and retracted, as in scirrhus, becomes distended and thinned, assuming a glistening or polished appearance. The transformations which takes place during the progress of the encephaloid cancer are dwelt upon at length, and the peculiar characteristics of the different species of ulcerations are carefully discriminated.

The chapter on *melanosis* or black cancer, is somewhat meagre, and no new facts concerning this much-disputed subject are elicited. The author believes melanotic cancer to be a form of the disease peculiar in its characters, and does not adopt the view hitherto so generally held, viz., that melanotic cancer is simply a form of the ordinary carcinoma, discolored as it were by the addition of pigmentary corpuscles.

The remaining pages of this first chapter are appropriated to the examination of some of the rarer forms of cancer, the chondroid and fibro-plastic cancers. It is in these tumors that so many of the fibro-plastic elements, mere elements of growth, occur; and as a general rule, they are not considered to be malignant.

Chapter second of the work is devoted to the study of the diagnosis of cancer. The distinction between the scirrhus and encephaloid varieties is thus described: "The scirrhus has always in it a something hard, the encephaloid a something soft; the encephaloid gives the idea of embossments (*bosselures*) tending to swell irregularly, to become prominent externally; in the scirrhus, on the contrary, the tumor and the integuments have a shrivelled, indurated look; the encephaloid distends, thins, reddens, and ulcerates the skin from within outward; the scirrhus seizes on the integuments by dragging them toward it, thickening them at first, corrugating or folding them, and seems to give rise to ulceration from without inward." The diagnosis between the different varieties of hard cancer is, however, according to the writer, more difficult, since some of them present to a great degree the same external characteristics; as for example, the chondroid and colloid cancer, and the simple innocent adenoid. The distinction between the encephaloid and melanotic tumors is at first easily made out; but as the former advances in its course, and assumes more and more the peculiarities of the fungus hæmatodes, embarrassment may arise; on the other hand, harmless tumors, infiltrated with carbonaceous or pigmentary matter, may at first sight be mistaken for melanosis.

The form of cancer which M. Velpeau denominates as keloid, is described as occurring only in the skin. He considers it, in fact, as a kind of transformed cicatrix, recognizable from ordinary scirrhus, from the fact that it occurs in patches raised above the level of the surrounding integument, in relief as it were. Its color is reddish, and its section perfectly homogeneous. It, moreover, never furnishes under pressure the so-called "cancerous juice." These facts serve, in the opinion of the author, always to distinguish this form of cancer from the true scirrhus; in which latter, should it involve the skin, there always exists a tendency to depression, or ulceration. It presents, in addition, a more unequal firmness, and furnishes besides a cancerous juice.

The second portion of this chapter treats of the diagnosis of cancer by means of the microscope. M. Velpeau passes in review the studies of microscopists during the last twenty years, and admits that the labors of Müller, Vogel, Virchow, Bennett, Lebert, Robin, and others, are sufficiently interesting, in a scientific point of view. He thinks, however, that as yet we have not realized results of a sufficiently *practical* nature to warrant our basing a diagnosis of malignant tumors simply upon a reference to the microscope. Researches by means of this instrument M. Velpeau does not forbid, for he adds: "By all means, let microscopic researches be called in to aid in the diagnosis of cancer; but in a clinical point of view they would lead to dangerous errors, if a real importance were acceded to them. . . . . The cancerous cellule exists, according to the evidence of microscopists themselves, in products which absolutely have nothing cancerous in them."

The chapter on the utility of the microscope as a means of diagnosis terminates with the following passage:

"We may say, then, of the microscope what I said of the pain,—it clears up nothing, and creates only doubt and uncertainty at the time when its aid is most wanted, in the first period of cancerous tumors; the diagnosis of the disease is sufficiently clear, on the contrary, when it is in a position to give an affirmative or negative evidence.

"Thus, I do not admit that it is as yet possible to diagnose a cancer of the breast by the microscope, better than by means of ordinary semeiology, and of purely clinical observation."

The nature and etiology of cancer form the subject of the third chapter. External violence *alone*, or repeated irritations, are not sufficient, in the judgment of M. Velpeau, to give rise to the development of cancerous growths. At the same time, the apparent influence exerted by external causes in the production of the disease is not entirely denied. In the category of tumors produced, perhaps, in this manner, we observe the so-called cancroid growths,—the epithelial cancers and tumors of most writers. Instances of carcinomatous affections, the result of the use of issues, blisters, and the application of caustic sulphuric acid, are also adduced in this connection.

In all these cases, however, the author seems to believe that a special predisposition to cancer has existed; and he is inclined to look upon external irritations simply as the occasional causes, without which, perhaps, the constitutional affection would not have been made manifest.

This predisposing cause M. Velpeau considers to be found "neither in the age, nor sex, nor in the constitution, nor in the general state of the health, nor in the climate, nor even in the nature of the tissues."

We must confess, that we derive but little information from the pages devoted to this subject, and indeed it seems as if we are forced to adopt the general conclusion, that the causes and origin of the disease are as yet utterly unknown.

The prognosis of cancer forms the subject of the next chapter. This we may dismiss in a few words. Cancer, unresisted, never disappears, and unless the disease be checked by therapeutical measures, death, in some form or other, must be the inevitable result.

The treatment of cancer, M. Velpeau then proceeds to consider. Internal medication, by means of purgatives, hemlock, arsenic, mercury, the iodides, quinia, and cod-liver oil, he passes in review, and with this conclusion:

"To express it in two words, the cancerous nature of the disease being fully established, no remedy, no internal treatment has been discovered equal to its cure."

The external application of ointments, plasters, pastes, and poultices, have led to results no less unfavorable. The use of compression, as a curative means, M. Velpeau believes to be inefficient; and he regards the cases of reported recoveries under its application as unfounded, and explains them away by reference to errors in diagnosis. This peculiar mode of rebutting testimony strikes us, more especially in other passages, as one of the most unpleasant features in the volume before us. Whilst we admit with pleasure M. Velpeau's well-known skill as a diagnostician, we still think that at times he claims even more than is his due, in bringing forward his own diagnosis as almost infallible, at the same time dwelling with so much stress upon the proneness of all others to err.

The question of the treatment to be attempted in cases of cancer is ushered in by the author in the following terms:

"Must we, therefore, renounce all medical treatment of cancers of the breast? This is not my opinion. Although nothing hitherto has succeeded in overcoming this frightful disease, one is not the less obliged to oppose some remedy to it, at least as a palliative, from the commencement to the end. Besides, all that I have said is applicable only to evident cancer. Now, for the practitioner who is not sure of his diagnosis, and for the periods and the forms of disease still admitting of doubt, there is evidently room not to leave the patients without treatment.

"If you have to do with a partial hard scirrhus, or a hard scirrhus *en masse;* with a pustular or plated scirrhus, disseminated or of the cuirass form; with an encephaloid, epithelial, melanic, chondroïd, or fibro-plastic cancer, reckon on nothing; palliatives alone can be employed. But if the diagnosis be uncertain, and the tumor seem to hold a middle place between a scirrhus and hypertrophy of the mamma, between scirrhus and old phlegmasic indurations, between the lardaceous scirrhus, whether partial or *en masse*, and simple lardaceous indurations, then we must act.

"Active treatment seems to me the more indicated in such cases, that I have seen the actual cure by treatment of tumors which considerably resemble scirrhus, presuming always that these were not cases of true scirrhus."

In the ensuing article M. Velpeau gives us a general outline of his own method of dealing with malignant tumors at their commencement, or with innocent tumors, in which the diagnosis is obscure. This treatment consists in the application of leeches in the neighborhood of the tumor, the use of soap or hemlock plaster, frictions with mercurial or iodine pomades, mucilaginous or alkaline baths, and the internal administration of iodide of potassium or cod-liver oil.

If the case in question be of an encephaloid character, in which ulceration has already taken place, saturnine and astringent lotions, and the internal use of narcotics will be clearly indicated.

In the pages devoted to the consideration of the curative treatment of cancer by surgical interference, the author again returns to the study of the nature and etiology of the disease.

Opposition to the operation, he states, is based upon three distinct reasons: first on observation; secondly, on theory; and, thirdly, on the microscopic nature of the evil.

Observation of the results of excision of these tumors, does not, in the opinion of the writer, militate against the idea of operation. M. Velpeau is satisfied from his own experience, that complete and radical cures do frequently take place after one or more operations. In support of this view, five cases are advanced occurring in the author's own practice. He is, therefore, not inclined to lean too much upon the opinions and statistics of Cruveilhier, Boyer, Scarpa, and others, who have made the matter of operation a subject of investigation, but whose conclusions differ from his own.

The theoretic objection is based upon the question, whether cancer at its commencement be a local or constitutional affection. On this point we have already expressed the author's view, although it seems to us that certain passages bearing upon this subject are hardly reconcilable with each other.

The third objection drawn from data furnished by the microscope, is one which M. Velpeau is not at present prepared to admit,—as he adds:

"Moreover, it is not indispensable that the microscope be called in to decide whether a tumor just removed be cancerous or not. Place on one hand the harmless, and on the other the malignant, I do not hesitate affirming, that, with the clinical ideas I have so long endeavored to popularize, an experienced practitioner will distinguish without difficulty the cancerous from the non-cancerous. On this point I appeal to micrographers themselves, and especially to MM. Lebert and Follin, who observed my practice for several years. Have they not seen me, a hundred times, at the hospital, declare the nature of such tumors, decide on what was cancerous and what not, before and after the operation; and in this respect has their microscope ever done more than confirm the diagnosis I had previously given? When I have positively affirmed the fact, have they ever found me wrong? A doubt in the diagnosis is only possible at the commencement of the disease; later, doubtful cases are rare. If the skilled clinical observer hesitates then, the information furnished by the microscope is not in itself of a kind to reassure him, nor to alarm, in regard to the operation.

"The question, then, of operating or not, cannot as yet be decided by the microscope. What may be conceded to it as yet is, that the presence of cancerous cellules, so called, in a tumor presenting in other respects the characters of cancer, is calculated to augment the fears of a relapse after the operation, as their absence would increase the confidence, if the tumor resembled in other respects the harmless tumors."

The arguments in favor of the operation are then summed up under the following heads:

"From all these difficulties, embarrassments, and from a crowd of other difficulties it were easy to accumulate here, it results, in my opinion:

"1st. That no plausible reason can be given for viewing cancer as a general disease, primarily.

"2d. That, on the contrary, it ought to be considered rather as a local disease in the first instance.

"3d. That certain tumors of a harmless nature, *seem* in some cases to undergo a malignant transformation.

"4th. That harmless or malignant tumors, adenoid, and even cancerous, of the breast, are probably caused by a plastic exudation, hematic or secreted, into the normal tissues, whether spontaneous, or exalted by external violence.

"5th. That the existence or the non-existence of the cancerous cellule in tumors, is no proof that the disease will or will not return after the operation.

"6th. That it would, therefore, be imprudent to trust to the microscope in deciding for or against the operation.

"7th. That observations and statistics are far from proving that the extirpation of tumors of the breast is always followed by relapse, always useless, or even injurious.

"8th. Finally, that facts sufficiently numerous prove, that observations drawn from my own practice demonstrate, without the possibility of doubt, radical cures by operation, of cancers the most unquestionable."

The alleged dangers of the operation are then discussed. These it is asserted are not serious except in the worst and most complicated cases.

Operation it is concluded is, 1st, "preferable to all other treatment against cancers, and even against adenoid or harmless tumors of the breast; 2d, it ought to be resorted to as early as possible." This rule of treatment is not, however, intended to apply to simple inflammatory indurations of the breast, or to hypertrophic swelling.

The author next passes, by a natural transition, to the mode of performing the operation. He prefers much to place the patient in a supine position, in order to prevent the occurrence of syncope. The incisions proper in each case must be governed by the size of the tumor, and the amount of tissue to be removed. Should accessory or secondary enlargements exist, either in the axilla or in the subpectoral hollow, the advice given is to remove them either by a fresh incision, or by an extension of the principal one. In removing axillary glands, it is advisable to tear them away by the fingers, rather than to separate them with the knife, in order to prevent increased and unnecessary effusion of blood.

The remaining portion of this chapter is occupied by the after-treatment of the operation. M. Velpeau is rather inclined to favor secondary reunion, or union by granulation.

Union by the first intention, although much to be desired, is nevertheless, he remarks, but rarely obtained throughout the entire extent of the wound. Hence there is always a tendency in the part to form sinuses or abscesses, liable to take on traumatic inflammation and erysipelas. The time requisite for complete cicatrization, either by the secondary or primary reunion, is about the same,—from five to six weeks. In all cases, therefore, of extensive tumors, in which a large portion of the integuments have been removed, M. Velpeau prefers the secondary union, or as he denominates it, the union by suppuration.

*Purulent infection*, following phlebitis, seems to be sufficiently rare as the result of amputations of the breast. The author reports but five well-marked examples out of two hundred and thirty-five operations, of which he has preserved notes. Erysipelas, on the other hand, appears to have been quite a frequent accident, the same record offering fifty-four instances of this complication. A special variety of erysipelas, which has hitherto excited but little attention, is that to which M. Velpeau applies the term "bronzed erysipelas."

This form of the disease evinces itself from the commencement, under the guise of brownish or bronzed plates. The skin, which is its seat, is thicker than under ordinary circumstances, and the erysipelas stands out in relief upon the surface.

"It is, as it were the ordinary erysipelas seen through a microscope. The plates are large from the beginning, and it is also accompanied from the first with a group of alarming symptoms. The pulse becomes very frequent, nausea often exists, the women are agitated, hot, tormented with thirst; they complain of anguish and suffocation; delirium comes on, and if life continues, gangrenous plates appear quickly at several points of the regions attacked with the erysipelas."

In the succeeding pages, the employment of caustics, as a curative means in the treatment of cancer, is examined. Premising that caustics have ever been the favorite agents of the quacks and empirics of all ages, M. Velpeau states that he has still thought it his duty to study their action. Since 1830 he has, therefore, despite the general anathema launched against them, submitted them to repeated trials in his practice at the hospital, and he concludes:

"It results from my experience that they ought not to be rejected absolutely, as a curative means. They are preferable to the cutting instrument:—1. When the cancer is ulcerated in plates, and broader than thick; 2. Whenever by the cutting instrument there is no room to preserve a part of the integuments involved by the tumor; 3. Whenever the cancer is fungous, exactly limited, and the patient greatly dreads the action of the bistoury; 4. Ulcerated scirrhus, anfractuous or disseminated, may be attacked with caustic more advantageously than with the bistoury; 5. It is the same with cancerous ulcers adhering to the summit of the axilla under the clavicle, in the neighborhood of bones.

"Beyond this, caustics ought not to be employed but at the special request of the patient or her family, and without forgetting that the facility of their application is the cause why they are recommended and strongly praised by a crowd of practitioners who dare not open a bistoury."

The special articles which have been employed are very various. Thus the butter of antimony, the Vienna paste, caustic potassa, sulphuric acid, the chloride of zinc, and arsenic, have each and all of them at different periods been greatly vaunted.

The sulphuric acid, and the chloride of zinc are the agents which in the hands of the author have yielded the best results. Arsenical preparations should be either eschewed, or else employed with great caution in consequence of their liability to become absorbed, and so to produce arsenical poisoning.

*Congelation* of cancerous masses, by means of a mixture of powdered ice and sea salt, as proposed by Mr. Arnott of Brighton, may, in the opinion of the author, hereafter furnish important beneficial results. He considers this agency as especially applicable to the destruction of cancerous plates, ulcerated tumors, vegetations, and fungosities. At the present moment, however, he believes congelation to be a "means still to be studied, rather than viewed as a means positively proved."

Dismissing the subject of active treatment, M. Velpeau next enters upon the consideration of one of the gravest questions connected with this terrible malady,—the liability to relapse after operation. The unfortunate fact is, we believe, admitted by every one, that science, as yet, has afforded mankind neither any preservative against cancer, nor any remedy which can prevent relapse. The development of secondary tumors can, in the opinion of our writer, be met simply by their removal, provided always, that the "tumor or tumors be movable, easy of extraction, and that the woman has no symptom of general infection." M. Velpeau believes that he has cured radically women after two or three successive operations.

The treatment of cancer is then concluded by a series of tables of scirrhus and encephaloid, from which, we confess, we cannot derive any very positive information. Those devoted to scirrhus strike us as extremely inaccurate: 146 cases only, are reported, whilst in the summary they are considered as amounting to 197. The enumeration of cases, by successive years, presents also apparently the same exaggeration. Of the 197

cases reported, 57 were not operated upon; whilst cauterization or operation was resorted to in the remaining 140. 70 out of the 140 continued well, or were in progress of cure when lost sight of; of the remaining 70, relapse had appeared in 22; 30 were dead, and of the remainder no mention is made. In the majority of cases reported in this table, no minute examination of the tumors had been made.

The statistics of encephaloid offer us 45 records of operations; of these 9 are dead,—of erysipelas, 3; of purulent infection, 3; of pleurisy, 2; of hospital gangrene, 1,—20 left cured; there was a relapse in 18; 18, had an ulcerated cancer. A cursory notice of the diseases of the mamma in man, and in new-born infants, and children, terminates a treatise in which a vast number of cases are collected and commented upon with characteristic ability.

If, indeed, we reflect upon the exalted position, the acknowledged skill in diagnosis, and the indomitable industry of the distinguished clinical teacher, a brief analysis of whose production we have just attempted, we cannot but feel glad, that these translations should afford to it a wider field of usefulness. The translations themselves are such as to reflect credit upon the writers, and that of the Sydenham Society particularly, is enriched, by the addition of many valuable notes upon the part of the editor, Mr. Henry.

In conclusion, if we were to express our opinion of the work before us, we would say, that it is essentially a practical one; the contained facts are well stated; but the deductions seem to us frequently to be given with a preformed opinion, and, certainly, sometimes with almost a degree of malice against the researches of modern minute pathology.—J. H. B.

